# Operando X-Ray Spectroscopic Techniques: A Focus on Hydrogen and Oxygen Evolution Reactions

**DOI:** 10.3389/fchem.2020.00023

**Published:** 2020-01-30

**Authors:** Varsha M. V, Gomathi Nageswaran

**Affiliations:** Indian Institute of Space Science and Technology, Thiruvananthapuram, India

**Keywords:** operando XPS, operando XAS, reactivity of electrocatalyst, hydrogen evolution (HER), oxygen evolution (OER)

## Abstract

The study of structural as well as chemical properties of an electrocatalyst in its reaction environment is a challenge in electrocatalysis. This is very important for the better understanding of the dynamic changes in the reactivity with respect to the structure of catalysts to give insight into the reaction mechanism. The *in situ/operando* investigation of electrode/electrolyte interface has been increasingly explored in recent days due to the significant developments in technology. The review focus on *operando* X-ray spectroscopic techniques to understand the behavior of electrocatalysts in hydrogen evolution and oxygen evolution reactions (HER and OER). Some recent studies on the application of *operando* X-ray spectroscopic methods to study the dynamic nature as well as the evaluation of structural and chemical changes of the electrocatalysts for HER and OER in different reaction environment are discussed.

## Introduction

Electrochemical reactions are a class of chemical reactions which can be initiated and controlled by electric current. The driving force for such type of reactions is the difference in energy between electrons in the electrode and the molecular orbital of chemical species in the solution (Elgrishi et al., 2017). The role of a catalyst in electrochemical reaction is to reduce the overpotential required for driving a reaction (Li and Li, 2017). The redox reactions that occur at the interface of electrode/electrolyte system through the direct electron transfer is responsible for the electrode overpotential. The electrode plays the role of an electron donor/acceptor as well as a catalyst, accelerating the rate of reaction at the interface of electrode/electrolyte. The study of the relation between structural and chemical properties of electrode materials and the mechanism of electrode reactions is very important in developing novel or improved electrocatalysts in various applications such as fuel cells (Zhang et al., 2010, 2013; Crumlin et al., 2012, 2013), batteries (Arthur et al., 2012; Liu et al., 2012, 2013, 2014), solar cells (Braun et al., 2012), etc.

The development of electrocatalysts depends on optimizing some key parameters which include activity, selectivity, stability, and yield (Bandarenka et al., 2014). The stability, conductivity, and the interactions at the electrode surface may change over time during a reaction. The activity of a catalyst material depends on certain parameters like its local geometry and the electronic structure which may change during a reaction (Lassalle-Kaiser et al., 2017a). So, in an electrochemical reaction, it is very important to understand whether the catalysts retain their chemical as well as structural properties throughout the reaction.

An electrochemical reaction can occur through different reaction pathways. The study of reaction kinetics is an important tool to obtain information regarding the active sites in a catalyst. However, it does not provide a clear idea about the exact reaction mechanism (Lukashuk and Foettinger, 2018). The use of operando spectroscopic techniques can overcome the limitations of kinetic methodology in understanding the reaction mechanism. It provides the information regarding the reaction mechanism, features of active sites and the structure of catalysts. This leads to a broader insight into the relation between structure and activity/selectivity of an electrocatalyst (Weckhuysen, 2003; Foster and Lobo, 2010).

The term *operando* (Latin word for working) refers to a class of analytical technique where a catalyst under operating conditions is monitored in real time and simultaneously characterizes its activity as well as selectivity. This approach allows to study the entire lifecycle of a catalyst from the preparation stage to its performance during reaction and its deactivation (Vogt and Weckhuysen, 2015). In the case of electrochemical reactions, due to the complex nature of catalyst surface, the presence of adsorbed species on the electrode surface, electrolyte, applied potential, etc., the use of operando techniques is quite challenging. However, compared to *ex situ* techniques, operando techniques provide deeper insight into basic mechanistic pathways behind the reactivity of electrocatalysts.

The study of electrocatalysts using *operando* techniques is increasing in the research field since the knowledge about the surface composition of electrocatalysts under reaction conditions obtained from *ex situ* techniques is limited (Chakrabarti et al., 2017). Most of the *ex situ* characterization techniques study the bulk of the catalysts. However, the bulk structure of a catalyst does not provide any idea regarding the surface composition (Karim et al., 2016). The variation of surface composition during reaction is taken into account to get accurate information regarding the nature of active site, deactivation mechanism, etc., which is essential for optimizing the catalyst design.

The operando techniques for the investigation of electrocatalysts include spectroscopic techniques like infrared (IR) spectroscopy, Raman spectroscopy, UV spectroscopy, X-ray absorption spectroscopy (XAS), X-ray photoelectron spectroscopy (XPS), and electron paramagnetic resonance (EPR) spectroscopy as well as microscopic techniques like atomic force microscopy (AFM) and transmission electron microscopy (TEM) (Lukashuk and Foettinger, 2018). The *in situ* use of these techniques for investigating catalytic processes under reaction conditions give information regarding the dynamic changes occurring on electrode surface. Table 1 gives a brief review of the operando techniques used for the study of different electrocatalysts/electrocatalytic reactions.

**Table 1:** Different operando techniques used for the study of electrocatalytic system.

**Operando technique**	**Electrocatalytic system**	**Parameters studied**	**References**
*In situ* Raman/SERS	Water oxidation	Reaction mechanism	Joya and Sala, 2015
*In situ*/operando Raman	Water splitting, CO_2_ reduction	Identification of catalytic active site, Elucidate reaction mechanism	Deng and Yeo, 2017
Operando Raman	CO_2_ reduction	Study of surface adsorbed species	Smith et al., 1997
Operando Raman and XAS	CO_2_ reduction	Identification of intermediates and structural changes	Dutta et al., 2018
*In situ* XAS, *in situ* Raman	Water oxidation	Reaction mechanism, identification of surface adsorbed species	Wang et al., 2016
Operando XAS	Ethanol oxidation	Study of structural changes	Melke et al., 2010
Operando XAS	O_2_ evolution	Identification of active site	Wang et al., 2015
Operando XAS	O_2_ reduction	Structure-activity correlation	Ziegelbauer et al., 2008
Operando XAS	Water splitting	Study of electronic and geometric structure	Fabbri et al., 2017
*In situ* XAS	Methanol oxidation	Reaction mechanism	Pelliccione et al., 2013
Operando XAFS	CO_2_ reduction	Structure-activity correlation	Genovese et al., 2018
*In situ*/operando IR spectroscopy	CO_2_ reduction	Monitoring the adsorbed species on catalyst surface	Handoko et al., 2018
Operando IR	CO_2_ reduction	Reaction mechanism	Firet and Smith, 2016
*In situ* IR	H_2_ oxidation	Study of catalytic active site	Hidalgo et al., 2015
*In situ* IR	CO reduction	Identification of intermediate	Pérez-Gallent et al., 2017
*In situ* IR	CO oxidation and adsorption	Reaction mechanism, structure of catalytic active site	Lebedeva et al., 2002
Mossbauer spectroscopy	Water oxidation	Identification of catalytic active site, oxidation state	Chen et al., 2015

In order to probe fundamental processes at the electrode/electrolyte interface, there are two different approaches used in operando techniques (Hartl et al., 2011; Bandarenka et al., 2014). The first approach involves applying conventional characterization methods like infrared spectroscopy, transmission electron microscopy, electrochemical scanning tunneling microscopy, near ambient pressure X-ray photoelectron spectroscopy, etc. Due to experimental constraints like low operating pressure, use of thin liquid layer and negligible current density, this mode of probing the interfaces is limited. The second approach is to make use of techniques which is considered as bulk sensitive such as X-ray diffraction (XRD) and XAS to study the electrode/electrolyte interface. It can be utilized to study the nanostructured electrocatalysts with high surface area that generate signal due to reactions at the interface. Both these techniques provide molecular level understanding of the electrode/electrolyte interfacial processes.

To understand the structural and chemical changes during reaction conditions and the electrochemical nature of catalysts, a number of operando techniques have been developed. Among different *operando* techniques, study of electrocatalysts using operando X-ray spectroscopies possess a significant place due to the potential of X-ray photons to perforate matter at even millimeter range. The focus of this review is first to review the *in situ*/operando XPS and XAS techniques, then a thorough examination of recent advances of its application to study the nature of electrocatalytic systems used for HER and OER.

## *In Situ*/Operando X-Ray Photoelectron Spectroscopy

**Figure 1:**
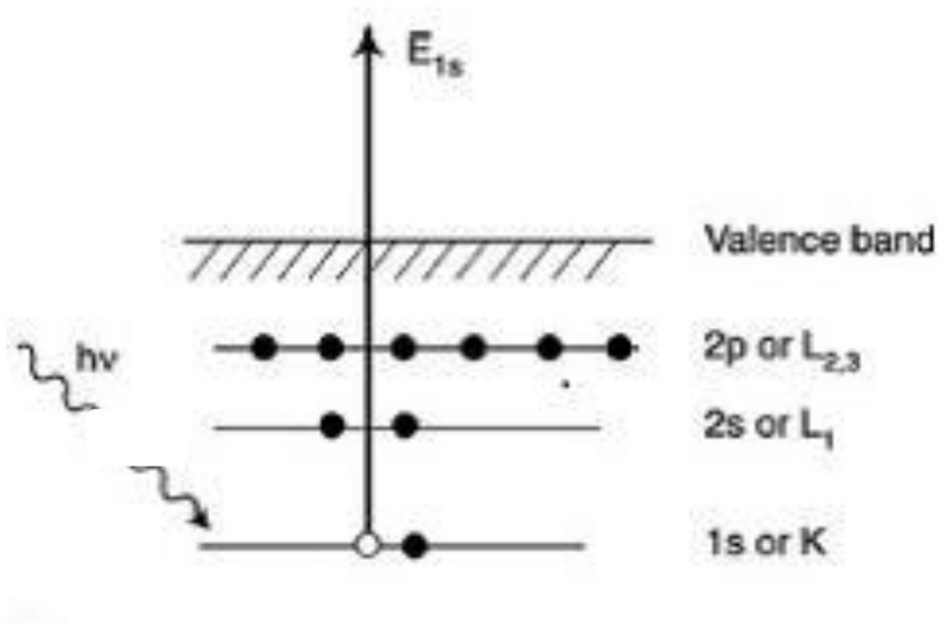
Schematic representation of XPS excitation process. Reproduced from Tougaard (2013) with permission from © 2013 Elsevier.

X-ray photoelectron spectroscopy (XPS) is a surface sensitive technique that require ultra-high vacuum (UHV) for operation (Crumlin et al., 2015). It measures the chemical and electronic state of elements within a material as well as its elemental composition (Ross, 1979; Hansen et al., 1980; Kolb et al., 1983; Kolb, 1987). The spectrum is obtained by irradiating the sample with X-ray beam (usually Al Kα or Mg Kα) and analyzing the kinetic energy of electrons ejected from the top 0 to 10 nm of the specimen. The irradiation of sample results in the ejection of core electrons which is analyzed using a detector (Figure 1). The photoelectrons emitted escape to the vacuum chamber and separated according to its energy. The electron analyzer and X-ray source require high vacuum conditions for operation. So, the conventional XPS is operated under UHV condition which limits its application for studying surfaces under ambient conditions (i.e., in presence of gases and liquids).

In the case of electrocatalysis, that requires ambient conditions, it is necessary to develop techniques for surface investigation of materials which is compatible with the given environment. There are certain challenges associated with using UHV-XPS under the electrochemical environment (Choi et al., 2017). The scattering of electrons from sample in gas or liquid environment can reduce the inelastic mean free path of electrons. Also, maintaining a liquid electrolyte in a UHV chamber is very difficult. In addition, the electron analyzer require UHV conditions to operate. These limitations can be tackled by coupling the electrochemical system with *in situ* XPS analysis.

The initial *in situ* study of electrochemical system using XPS were carried out at UHV (10^−9^ Torr) or near UHV conditions (10^−6^ Torr) (Crumlin et al., 2013). The further improvement of XPS is with the introduction of vacuum sealed electrochemical cell covered with X-ray transparent window which enables the study of liquid electrochemical system (Bozzini et al., 2008, 2009, 2010, 2011; Gianoncelli et al., 2011). In addition, the aperture size is minimized with respect to analyzer lens system and a design of differential pumping is introduced before the analyzer (Ogletree et al., 2009; Starr et al., 2013). In order to tackle the liquid electrolyte problem, an electrode is dipped into an electrolyte in a beaker inside the XPS chamber. After the thin layer formation of an electrolyte across the surface of electrode, the electrolyte containing beaker can be removed (“dip and pull” method). The operating pressure is of the order of Torr and it is called ambient pressure X-ray photoelectron spectroscopy (APXPS).

The development of APXPS instrument has gone through a number of stages. Initially, APXPS instruments having lab-based X-ray sources were used (Joyner et al., 1979; Siegbahn et al., 1981; Siegbahn, 1985; Ruppender et al., 1990; Bukhtiyarov et al., 2005; Pantförder et al., 2005). The application of improved XPS system using synchrotron X-ray sources started since 2000. The design of differentially-pumped electrostatic pre-lens, that separate the sample chamber from an electron analyzer exhibited increased photoelectron detection efficiency up to three orders of magnitude (Ogletree et al., 2002, 2009). Presently, commercially available APXPS electron analyzers are equipped with synchrotron based X-ray sources (Salmeron and Schlögl, 2008; Bluhm, 2010; Schnadt et al., 2012).

The introduction of synchrotron radiation sources that provide X- rays with high brightness paved the way for exploring new aspects of APXPS (Salmeron and Schlögl, 2008; Kaya et al., 2013). The small cross section of electron beam make it as a very bright source which resulted in high resolution imaging of the sample (Salmeron and Schlögl, 2008). Synchrotron radiation sources provide very high photon flux than conventional sources which is of several orders of magnitude. They can probe the structure of materials from sub-nanometer electronic structure level to micro or millimeter level. The characteristic features of synchrotron radiation include high brilliance, high polarization level, low angular divergence of the beam and pulsed emission of light. An advantage of synchrotron technique is the rapid data acquisition that allows time resolved collection of data. This give insights into processes in real time. The energy of synchrotron X-ray photons can be tuned easily over a broad spectrum. This resulted in a number of different ambient pressure XPS methods. The binding energy of core-level electrons in an atom is probed using APXPS. The most surface sensitive approach involves the use of photons of lower energy (<1 keV) that produce photoelectrons of smaller kinetic energy (~100 eV) (Ogletree et al., 2002; Starr et al., 2013). The non-destructive study of interfaces is possible by the use of photons in the energy range of 2–6 keV. Finally, the use of high energy X-rays (>6 keV) enables deeper insight into the bulk of materials (Fadley, 2005).

The development of electron pre-lens, differential pumping, and the design of sample atmosphere leads to the abrupt growth of research on the solid/gas and solid/liquid interfaces using synchrotron-based APXPS (Starr et al., 2013). The study of electrochemical solid/gas interface using *operando* APXPS begins with its application in Solid Oxide Electrochemical Cell (SOEC) (Crumlin et al., 2013; Liu et al., 2014). The ease of operation in gas environments and the use of soft X-rays make them suitable for studying the properties of solid/gas interface. It consists of specially built cells that uncover the interface of electrode/electrolyte to a gaseous environment and simultaneously exposing to the X-ray source. The electron analyzer is connected to a potentiostat to provide an applied voltage (Crumlin et al., 2015).

## *In Situ*/Operando X-Ray Absorption Spectroscopy (XAS)

XAS is a core level spectroscopy where the incoming X-rays interact with the core electrons in an atom. It is an efficient tool to probe the electronic structure of materials. Hard X-rays (>5 keV) probe the chemical and physical properties of energy related materials including batteries and fuel cells. Soft X-rays (<3 keV), that probe a shallow depth, requires stringent vacuum condition and is not much explored in materials research. It has limited the application of soft X-ray spectroscopy on systems under *in situ*/*operando* conditions.

**Figure 2:**
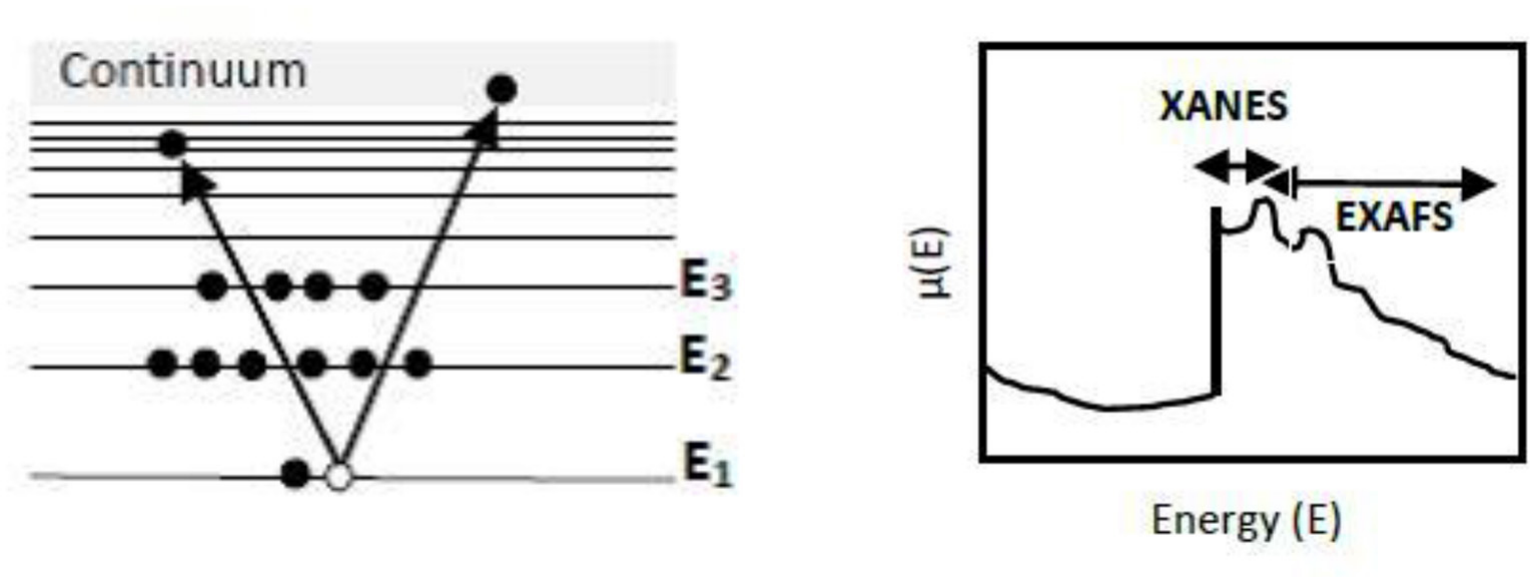
**(A)** Schematic diagram of XAS absorption and **(B)** Absorption coefficient μ*(E)* vs. photon energy *(E)* with the fine structure. Adapted from Schnohr and Ridgway (2015).

In an XAS measurement, when the sample is irradiated with X-rays, the core electron gets excited to an unoccupied energy state. This results in an increase in the absorption coefficient (Figure 2A). Thus, XAS gives information regarding the unoccupied electronic structure of an atom. This increment in absorption at a given incident energy corresponds to the energy difference between the core level and unoccupied bound state. Further, if the X-rays have sufficiently higher energy (>30 eV), the photoelectron gets excited to continuum. The higher energy electron decays to the ground state by emitting a photon, i.e., XAS is a “photon in-photon out” process. The XAS spectrum consists of two regions, namely the XANES (X-ray absorption near-edge structure) and EXAFS (Extended X-ray absorption fine-structure) as shown in Figure 2B (Schnohr and Ridgway, 2015).

The techniques like EXAFS and XANES are used to study the internal structure of nanoparticles and the species adsorbed on them (Choi et al., 2017). XANES measurements are carried out in the vicinity of the absorption edge. This region is characterized by the excitation of core electron to an unoccupied state. Therefore, this region gives information regarding the chemical bonding as well as the oxidation state of the absorbing atom. It also gives details about the symmetry of metal site, local environment of atom, structural disorder of absorbing atom. The multiple scattering phenomena in XANES that depends on the geometry of crystalline material enables to differentiate among different crystalline phases (Bunker, 2010). The XANES region is also pointed out as near edge X-ray absorption fine structure (NEXAFS). EXAFS is widely used for the study of disordered and amorphous materials including heterogeneous catalysts. It resulted from the excitement of photoelectrons to the continuum state. It can probe the local, short-range coordination domain of atoms in an electrocatalyst and provide information regarding the electronic structure of specific sites in a material, interatomic distance, coordination number, etc. (Rehr and Albers, 2000).

XAS measurements can be performed under realistic working conditions unlike XPS that require UHV atmosphere for operation. Also, hard (wavelengths down to 10 pm) or soft X-rays (wavelength up to 10 nm) can be used to probe the catalyst material (Choi et al., 2017). The simple operando cell used for XAS consists of a thin electrolyte layer in between an anode and cathode placed parallel. A drawback of this setup is the absorption and diminishment of the incident radiation in the liquid electrolyte. In order to overcome this limitation, catalyst is held on an X-ray transparent, conducting film that acts as an X-ray window. This eliminates the attenuation of X-rays by the electrolyte. The changes in the structure of working catalysts can be monitored *in situ* using operando XANES and EXAFS.

**Figure 3:**
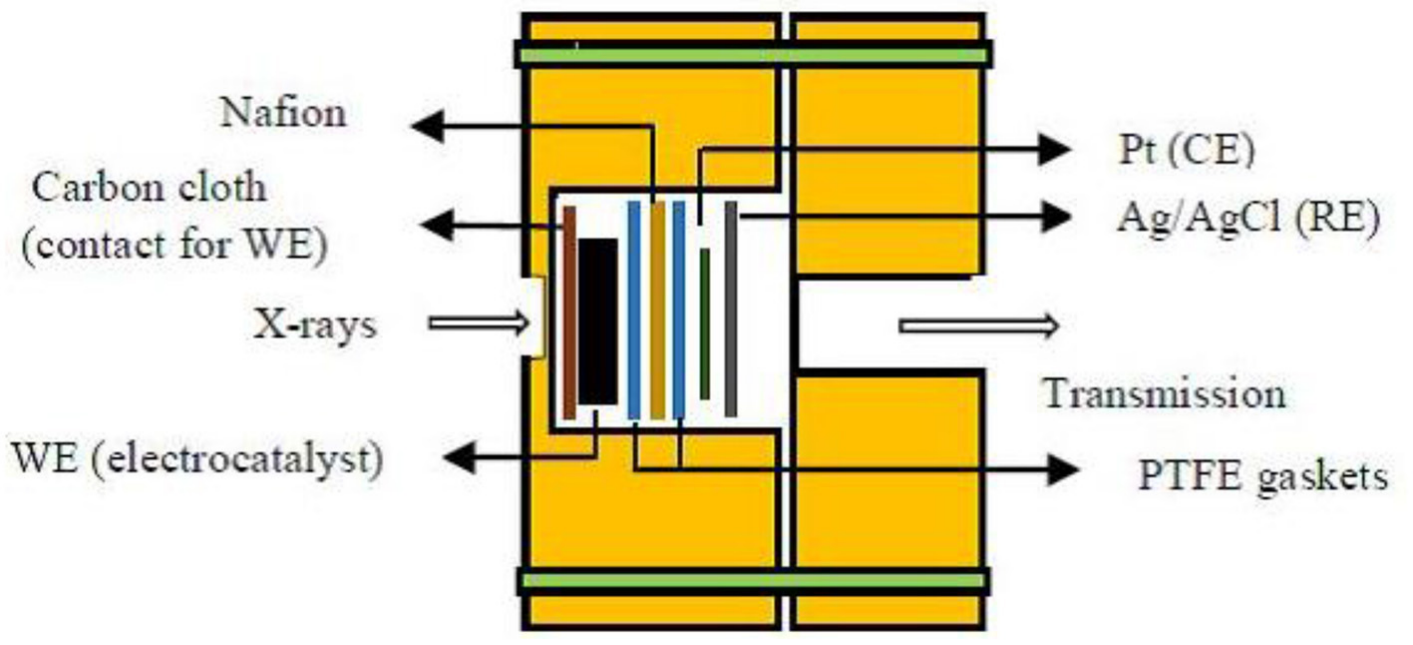
Schematic diagram of *in situ* electrochemical cell used for X-ray absorption spectroscopy. Adapted from Sasaki et al. (2010).

Figure 3 represents the schematic of an *in situ* electrochemical cell for XAS experiments used by Sasaki et al. (2010). The working catalyst (Pt/Pd/C), nafion (proton exchange membrane), and two PTFE gaskets were sandwiched. The cell components were strongly held by acrylic plastic bodies having transparent X-ray windows. Pt foil and Ag/AgCl act as the counter and reference electrodes (CE and RE), respectively. This electrochemical cell setup is designed in such a way that it can acquire data in both transmission and fluorescence modes.

The absorption is measured by the attenuation of incident X-rays, whereas soft X-rays capable of penetrating shallow depth limits the measurement of transmission to ultra-thin films. XAS measure the intensity of core-hole decay products, i.e., the total yield of fluorescence photons (TFY) and total electron yield (TEY) or Auger electrons. Both TFY and TEY signals provide information about the density of unoccupied electronic states which are proportional to the number of holes created in the core-level. There will be a change in probe depth between TEY and TFY (Crumlin et al., 2015). This difference is used for understanding the difference between bulk and surface state of a sample.

**Figure 4:**
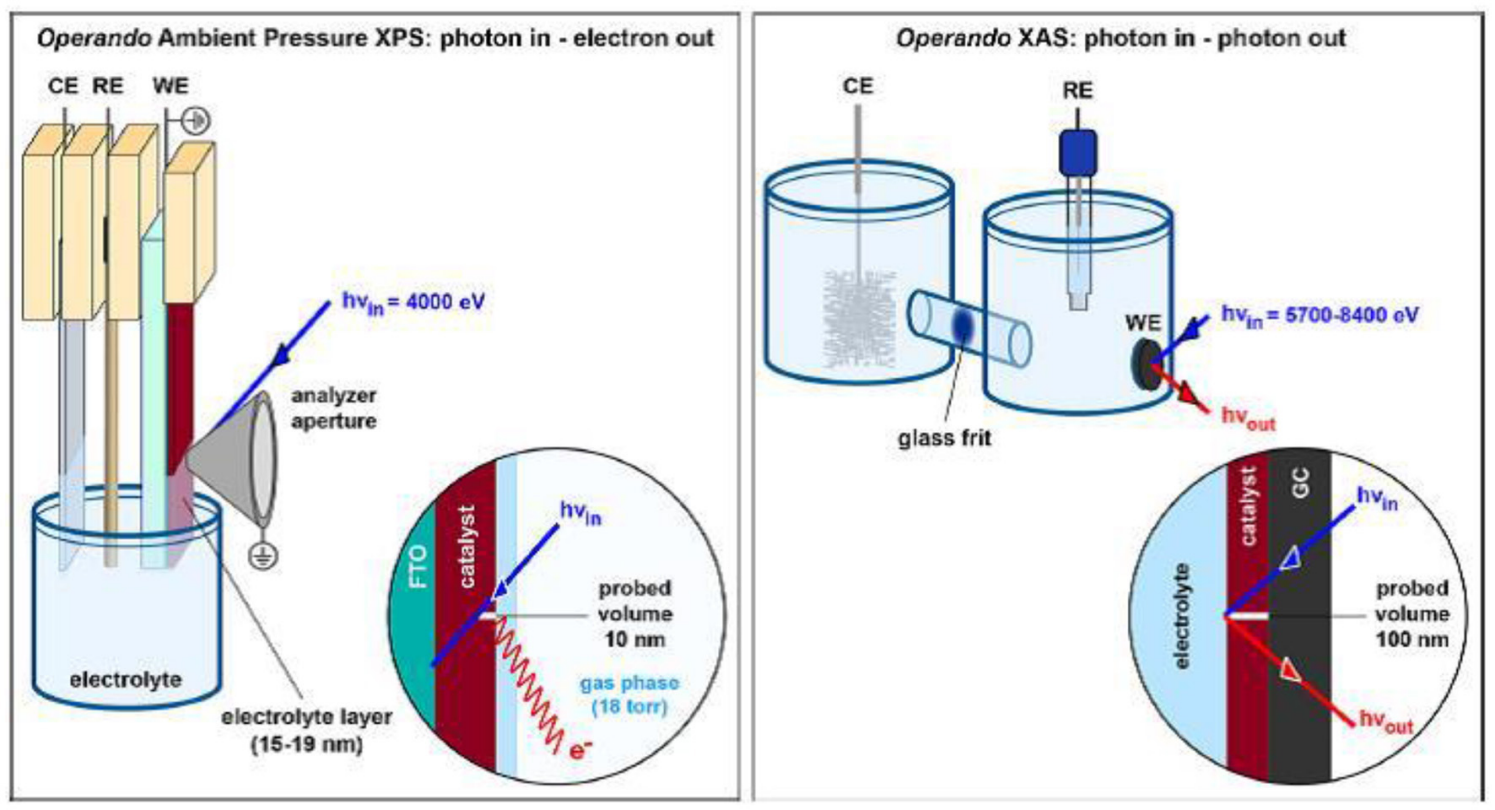
Schematic diagram of APXPS and XAS. The detection modes of the two techniques and the corresponding probed volume are given in the inset. Reproduced from Favaro et al. (2017a) with permission from © 2017 American Chemical Society.

Both the techniques probe different volumes of the catalyst upon interaction under operating conditions as depicted in Figure 4. XPS and XAS analyze photoelectrons and fluoresced photons, respectively. In XPS analysis, the sampling depth depends on the energy of incident X-rays and probe the surface up to 10 nm. Most of the XPS measurements are done with tender X-rays since the effective attenuation length of ejected electrons is higher than that with soft X-rays (Favaro et al., 2017a,b). This enables the study of sample surface covered with electrolyte layer of few nm thickness. On the other hand, the use of XAS analysis with hard X-rays probe the sample surface up to few micron range. The coupling of two techniques provide information regarding surface chemistry as well as the coordination environment of atoms throughout the sample.

## Electrochemical Processes for the Production of Fuel

The global demand for energy is increasing in parallel with socio-economic development. The current energy requirements are largely met by non-renewable fossil fuels that is depleting in an alarming rate. Therefore, the use of renewable sources to meet energy demands is widely explored. The energy systems like fuel cell, metal-air batteries, solar cells, etc., which is based on electrochemical reactions are simple, efficient, and reliable class of techniques (Tahir et al., 2017). The evolution of hydrogen and/or oxygen are the core reactions in these systems.

**Figure 5:**
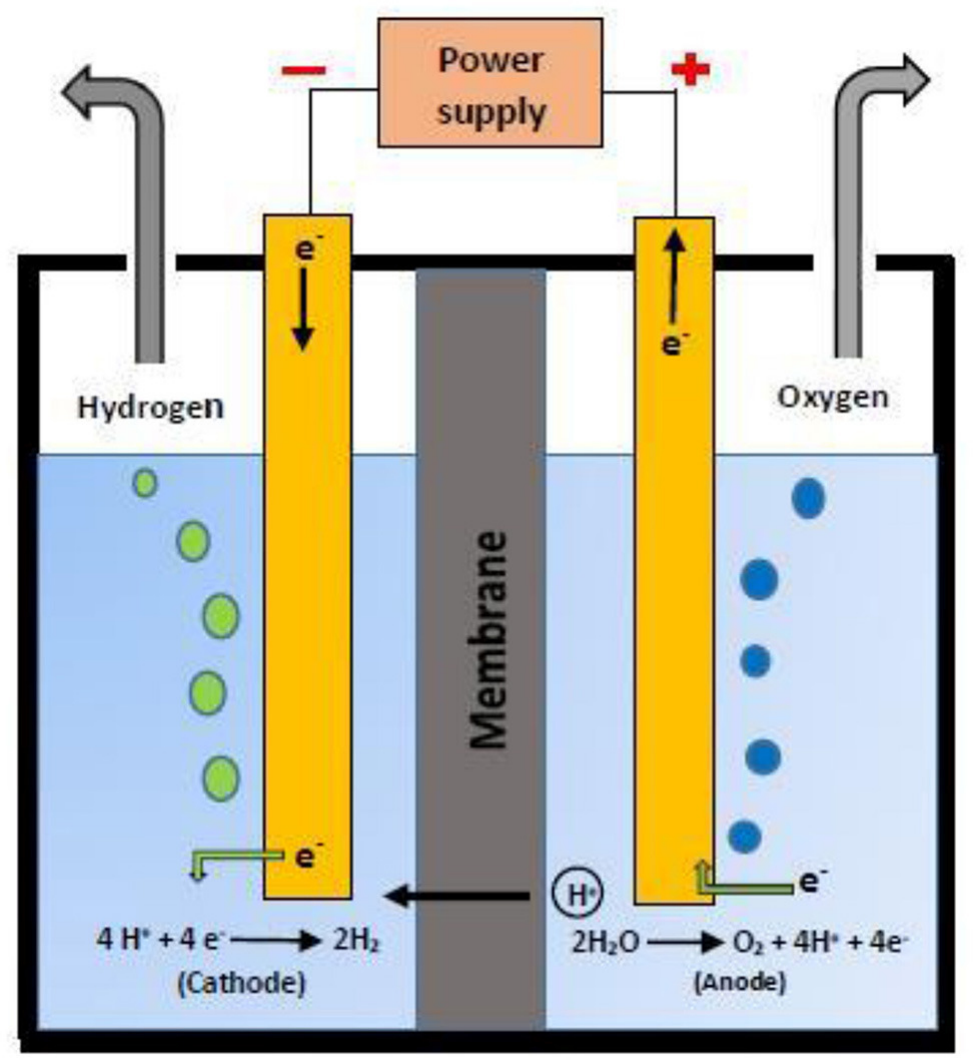
Schematic diagram of Electrolyzer for water splitting.

Water splitting is a process that enables the decomposition of water into its constituent elements i.e., hydrogen and oxygen (Figure 5). The energy required to cleave the H-O-H bond is provided by different energy sources like electric current, thermal and light. Based on these energy sources, water splitting process is classified into electrolysis, thermolysis, and photolysis, respectively (Albonetti et al., 2019). In electrolytic water splitting, the passage of electric current through water results in the formation of oxygen at anode and hydrogen evolution at cathode in a unit called electrolyzer (Figure 5). The mechanism of electrolytic water splitting can be given as (Bockris and Potter, 1952; Penner, 2006):

$$\begin{array}{l} \;\text{At cathode }\left(\text{reduction}\right):4{\text{H}}^{+}+4{\text{e}}^{-}\to 2{\text{H}}_{2}\text{ E}=0.00\text{Vvs}.\text{RHE}\\ \text{At anode }\left(\text{oxidation}\right):2{\text{H}}_{2}\text{O}\to {\text{O}}_{2}+4{\text{H}}^{+}+4{\text{e}}^{-}\text{ E}=+1.23\text{Vvs}.\text{ RHE}\\ \;\text{Overall reaction}:2{\text{H}}_{2}\text{O}\to 2{\text{H}}_{2}+{\text{O}}_{2}\;\Delta \text{E}=-1.23\text{ V} \end{array}$$

In order to produce hydrogen, electrochemical splitting of water is regarded as a renewable, clean, and efficient method (Kibsgaard and Jaramillo, 2014). It is considered as an environmental friendly technique with zero emission of CO_2_ since oxygen is the only by-product (Du and Eisenberg, 2012; Narayan et al., 2015; Ma et al., 2016). Therefore, a great amount of research is oriented in the field of electrochemical/catalytic water splitting reaction. In the field of energy research, the development of highly efficient and economically feasible electrocatalysts for hydrogen and oxygen evolution reactions is very important.

Hydrogen is considered as a suitable candidate for environmental friendly fuel due to its high energy density (Wang and Gao, 2018). Hydrogen evolution reaction (HER) being one of the most commonly studied electrochemical process is a sustainable source of hydrogen in the fuel cells. The overpotential necessary to drive the HER can be minimized by the suitable choice of an electrocatalyst. The oxygen evolution reaction (OER) or water oxidation is a multi-step process which involves the transfer of four electrons with transfer of one-electron at each step. The energy involved in each step varies significantly. Also, the reaction is highly dependent on the pH of the reaction medium. In acidic and neutral conditions, an applied voltage of about 1.23 V and in basic environment a potential of 0.404 V is required for water splitting. The equilibrium half-cell reaction is given below:

$$\begin{array}{l} 4\text{O}{\text{H}}^{-}\to 2{\text{H}}_{2}\text{O}+{\text{O}}_{2}+4{\text{e}}^{-}\;\left(\text{Inalkaline medium}\right)\\ \;2{\text{H}}_{2}\text{O}\to 4{\text{H}}^{+}+{\text{O}}_{2}+4{\text{e}}^{-}\;\left(\text{Inacidic medium}\right) \end{array}$$

The variation in the energy at each step of reaction as well as pH dependence makes the kinetics of OER sluggish and leads to higher overpotential. Therefore, stable and highly active electrocatalyst is required to overcome the energy barrier for the reaction (Gorlin and Jaramillo, 2010; Gong et al., 2013; Tahir et al., 2015b).

Fuel cell transforms the chemical energy stored in a fuel by catalytic reaction into electrical energy (Tahir et al., 2015a; Zhang et al., 2015; O'hayre et al., 2016). In a simple fuel cell, electrochemical half reactions occur on respective electrodes which is separated by a suitable electrolyte by consuming the fuel supplied. H_2_-O_2_ fuel cell is the common example of a reversible fuel cell where hydrogen and oxygen are used as the fuel. The half-cell reactions can be given as:

$$\begin{array}{l} \;{\text{H}}_{2}\to 2{\text{H}}^{+}+\;2{\text{e}}^{-}\\ ½{\text{O}}_{2}+2{\text{H}}^{+}+2{\text{e}}^{-}\to {\text{H}}_{2}\text{O} \end{array}$$

In the first phase of electrocatalysis in fuel cell, H_2_ and O_2_ are produced by the electrolytic splitting of water (Saadi et al., 2013). HER and OER are the basic chemical reactions that produce the fuels. The commercialization of fuel cell is facing difficulties due to the higher cost, poor efficiency and stability of electrocatalysts used in anodic and cathodic reactions in fuel cell. This shows the significance of development of suitable catalysts that can be used in fuel cells for the *in situ* water electrocatalysis that provide hydrogen and oxygen fuels (Tahir et al., 2017).

A recent study on the electrochemical oxidation of Nickel utilized the APXPS to obtain depth resolved information about the chemical species (Karslioglu et al., 2015). They have investigated the complete oxidation/hydroxylation of Ni film of 8 mm thickness in the presence of 0.1 M KOH solution at an applied potential of +0.6 V. In another study, a closed cell of liquid electrolyte sealed by a very thin silicon film is developed. Here, the solid/liquid interface is probed by the hard X-rays generated in the vacuum side that allows for the real time monitoring of electrochemical conditions at the interface (Masuda et al., 2013). The *in situ* solid/gas APXPS measurement provide information regarding the work function and the properties of a catalyst surface under operating condition. This technique can also be applied when the catalytic material undergo reaction in presence of different reacting gases and varying temperature.

## *In Situ*/Operando XPS Studies on Her Electrocatalysts

Casalongue et al. designed an electrochemical cell compatible with APXPS (Casalongue et al., 2013; Sanchez Casalongue et al., 2014) in which the working electrode (WE) was modified with amorphous molybdenum sulfide (MoS_3_) nanoparticles (Casalongue et al., 2014). The XPS spectra of MoS_3_ cathode was obtained using incident X-ray photon of energy 900 eV. The S 2p spectra of amorphous molybdenum sulfide (MoS_3_) NPs under various experimental conditions (like different water vapor pressure and applied cell voltage) obtained was compared with the S 2p spectra of crystalline MoS_2_ electrode. The earlier one shows increasing catalytic current density over time. Also, the increase in peak intensity at about 162 eV which is characteristic of MoS_2_ confirms that the amorphous MoS_3_ sites get reduced to MoS_2_ during reaction conditions. From the S 2p deconvoluted spectra before and after hydrogen evolution it is further confirmed that there is a notable increase in the MoS_2_ species. This is responsible for the increased current density coming from the cell. In short, there is a high interconnection between the current density obtained from electrochemical measurements and the chemical composition of HER catalyst observed from APXPS.

Stoerzinger et al. (2017) applied APXPS to investigate the effect of applied potential on oxidation/reduction of platinum species on polycrystalline Pt WE in alkaline electrolyte. The surface chemistry of WE and the changes in the Pt adsorbate species under a constant electrode potential was studied by characterizing the surface species using APXPS (hν = 4 kev) (Axnanda et al., 2015). The chemical composition of Pt WE was obtained from the Pt 4f core level spectra. The peaks corresponding to Pt^0^ metal, Pt-OH, Pt-H, Pt^(II)^O and Pt^(IV)^O_2_ species were obtained. The complex nature of the surface of Pt electrocatalyst during electrochemical polarization in alkaline medium is studied in *operando*. This study highlights the importance of operando techniques to generate new kinetic models for electrode/electrolyte interfaces under reaction conditions.

## *In Situ*/Operando XPS Studies on Oer Electrocatalysts

The *in situ* study of OER electrocatalysts with surface sensitive XPS technique provides insight into the phenomena occurring at the electrode/electrolyte interface (Arrigo et al., 2013; Axnanda et al., 2015; Law et al., 2015). The catalysts based on cobalt oxides (CoO_x_) exhibit desirable OER activity over a range of pH values (2–6) (Chen et al., 2009; Price et al., 2011; Trotochaud et al., 2012; Yang et al., 2014). Favaro et al. studied the structural and chemical evolution of a highly active biphasic CoO_x_ catalyst on silicon (Si) by operando APXPS using “tender” X-rays (E = 4.0 keV) (Favaro et al., 2017c). The operando measurements were performed using a three-electrode configuration in which WE coated with CoO_x_ is studied under OER conditions in a H_2_O-saturated reduced pressure environment (~18 Torr). Using the “dip and pull” method (Axnanda et al., 2015; Lichterman et al., 2015; Favaro et al., 2017b), a thin (~nm) electrolyte layer of 1.0 M KOH was formed on the WE surface in the analysis chamber. From the Co 2p, O 1s and valence band operando XPS analysis, the nature of catalyst surface was studied. From the Co 2p region the electronic and chemical state of CoO_x_ film was encoded. The double layer structure of CoO_x_ film consisting a Co(OH)_2_ (Co^2+^) layer on top of a Co_3_O_4_ (Co^2+^, Co^3+^) layer is confirmed from the deconvoluted Co 2p3/2 core level spectra under vacuum (in the absence of electrolyte) as well as hydrated conditions (presence of electrolyte). The comparison of spectral data with that of spinel Co_3_O_4_ catalyst shows that the presence of Co(OH)_2_ improves catalytic activity by improving the transition to CoO(OH).

The application of APXPS using “tender” X-rays to analyze a system under electrochemical conditions is reported by Ali-LöYtty et al. (2016). They have characterized Ni-Fe oxyhydroxide thin film of OER catalyst electrodeposited on gold at different voltage. The operando measurements of the chemical nature of catalyst coated with a ~30 nm 0.1 M KOH electrolyte layer based on applied potential above the onset potential of OER is carried out. There is a shift in binding energy observed for O 1s spectrum of water in gas and liquid phase as well as in the core level spectra of Ni and Fe. Further XPS analysis shows that, Ni and Fe species in the electrocatalyst are present in both metallic and oxidized state. During electrochemical reaction, metallic Fe and Ni get oxidized to Fe^3+^ and Ni^2+/3+^, respectively. Also, an increase in the O/OH ratio in the potential range of 0–0.3 V is observed which is due to the oxidation of Ni(OH)_2_ to NiOOH. In short, the nature of oxygen containing species present on the surface of catalyst was analyzed *in situ*.

## *In Situ*/Operando XAS Studies on Her Electrocatalysts

The origin of catalytic activity of an amorphous cobalt sulfide (CoS_x_) HER catalyst was investigated through *operando* XAS analysis. The CoS_x_ catalyst immersed in electrolyte in open circuit condition was analyzed under *operando* and compared with the dry sample. CoS_x_ under HER operating conditions exhibited a transient behavior where the clusters of cobalt oxide and sulfide instantaneously converted into CoS_2_ like structures with high density of sulfur active sites (Kornienko et al., 2015). Fadl et al. investigated electrodeposited cobalt phosphide (CoP) films HER electrocatalyst under operando condition using Co K-edge and P K-edge XAS (Saadi et al., 2017). It consists of analysis using XANES and EXAFS data. From the analysis it is observed that the electrocatalyst is amorphous in nature and consists of near-zero valent Co as well as reduced P. B Lassalle et al. studied the structural evolution of the amorphous cobalt nanoparticles under working condition using operando Co K-edge XANES. The cobalt precursor complex solution was taken in a spectroelectrochemical cell with glassy carbon plate as the WE. At a constant potential of −0.3 V vs. RHE, Co NPs get deposited at the surface of glassy carbon. This electrodeposition process was monitored *in situ* using XANES. The amorphous nature of electrocatalyst is confirmed from EXAFS (Lassalle-Kaiser et al., 2017b). Using operando X-ray absorption fine structure (XAFS), Cao et al. examined the dynamic electronic structure and local coordination environment of cobalt single atomic site (SAS) catalysts during alkaline HER (Cao et al., 2019). The nature and evolution of Co active site during HER condition is investigated using an operando electrochemical cell set up. It is obtained that the structure of SAS catalyst change drastically under reaction conditions. The adsorption of H_2_O on Co sites during HER is monitored using *operando* method.

Ni et al. (2019) developed a hydrogen oxidation reaction (HOR) catalyst in alkaline medium based on Ni_3_N. It also exhibits activity similar to Pt for HER in alkaline medium. *In situ* XAS studies revealed that Ni_3_N/C is a stable catalyst in the applied bias of 0–0.25 V vs. RHE. Chu et al. (2019) investigated the transformation of pyrite-phase Ni based electrocatalysts during HER using *in situ* XAS. The comparison of HER activity between isostructural elctrocatalysts like NiP_2_, Se-doped NiP_2_ and NiSe_2_ revealed that Se doped NiP_2_ has the highest activity. The local structure and valence state of catalysts were obtained from *in situ* studies. From XANES data, it is observed that the energy of absorption is higher for NiP_2_ suggesting the valence state is of the order of NiP_2_ > NiP_1.92_Se_0.08_ > NiSe_2_ and lies between Ni(0) and Ni(II). Also, the dynamic transition within each material is investigated using Ni K-edge XAS spectra obtained in 1 M KOH electrolyte. This suggested a change in the electronic structure of NiP_2_ from the initial condition during HER.

## *In Situ*/Operando XAS Studies on Oer Electrocatalysts

Gorlin et al. (2013) studied OER using a manganese oxide (MnO_x_) based bifunctional catalyst. A thin film of MnO_x_ electrodeposited on silicon nitride acts as the electrocatalyst (WE). The reaction is carried out with carbon as CE, Ag/AgCl as RE in 0.1 M KOH electrolyte. The *in situ* XAS analysis is performed at an onset potential of 1.8 ± 0.001 V. XANES analysis shows that MnO_x_ phase is more oxidized than the α-${\text{Mn}}_{2}^{\text{III}}$O_3_ phase and more reduced than the β-Mn^IV^O_2_ phase. From both XANES and EXAFS studies, it is observed that about 80% of the film get oxidized to generate a mixed Mn^III,IV^ oxide and about 20% of the film consisted of less oxidized phase (${\text{Mn}}_{3}^{\text{II},\text{III},\text{III}}$O_4_). The increased catalytic activity is due to the Mn^III,IV^ oxide phase. The presence of different phases is in accordance with the XPS results which shows two different oxidation state for the catalyst surface. In another study of electrodeposited OER catalyst by Friebel et al., operando XAS is used to examine the electronic environment of Fe doped NiO_x_ OER catalyst at different Ni and Fe ratios. Below the onset OER potential, in the absence of Fe, the Ni is present in α-Ni(OH)_2_ phase and as a double hydroxide structure in the presence of Fe.

There are studies reported on modified conventional *in situ* X-ray techniques to understand the interaction between metal ions in catalyst and the reactants. Hung et al. (2018) developed a high energy resolution fluorescence detected operando XAS equipped with small angle of incidence of X-ray that increase the interaction near the outer surface of catalyst. Fe doped binary oxide grown on carbon cloth was the electrocatalyst under investigation for OER. The operando studies conclude that the presence of Fe ions in the neighborhood of Co ions having higher oxidation states could stabilize the Co ions and leads to an efficient pathway for OER. Also, the Co ions present in the Fe doped spinel exhibited intense peak due to the interaction with the oxygen mediated reactant by *d*-*p* orbital hybridization during increased applied voltage of OER. This concluded that Co ions in the composite act as the prominent active site rather than Fe ions and Fe doped Co ion sites exhibited enhanced catalytic activity than that of Fe ion sites.

There are also reports on the operando investigation of bifunctional catalysts for both HER and OER under electrochemical conditions. In a recent work, Zhu et al. studied the chemical as well as structural stability of a bifuctional electrocatalyst based on phosphorous (P)- substituted CoSe_2_ in an alkaline electrolyte (1 M KOH) toward HER and OER (Zhu et al., 2019). The increase in the degree of phosphorization of CoSe_2_ resulted in a phase transition from cubic (*c*) CoSe_2_ to orthorhombic (*o*) CoSe_1.26_ P_1.42_. *In situ* Co K-edge XANES spectroscopic analysis was performed to understand the effect of phosphorous content in the transformation of these catalysts during reaction. From spectra it is observed that, for HER, the increase in cathodic potential to −0.36 V (vs. RHE), the *o*-CoSe_1.26_P_1.42_ catalyst compared with *c*-CoSe2 has oxidation state closer to that of Co foil. This observation concluded the increased catalytic activity of o-CoSe_1.26_P_1.42_ to the near metallic state of Cobalt, i.e., the metallic state of Co act as the reactive species. Whereas, in the case of OER, with the increase in anodic potential, both *c*-CoSe_2_ and *o*-CoSe_1.26_P_1.42_ exist in higher oxidation state as confirmed from the increase in the spectral intensity and shift toward higher energy in the XANES spectrum. The introduction of P resulted in a chemical state change to cobalt oxyhydroxide that act as the reactive species for OER. In short, *in situ* spectral analysis concludes that the incorporation of P to CoSe_2_ triggers the structural transformation from the precatalytic state to real reactive species.

## Conclusion

In this brief review, we have explained the electrochemical systems for HER and OER catalysis, which are investigated using *operando* X-ray spectroscopies. *Operando* techniques can be used to analyze the structural as well as chemical changes of electrocatalysts under working environment which give insight into the mechanistic pathway of a reaction. The structural phase of amorphous materials, change in composition during reaction and the oxidation state can be determined as a function of the applied voltage. This knowledge can be utilized to propose reaction mechanism and explain the merits and drawbacks of a catalyst. The probing of the surface of a catalyst using *operando* methods enables to explore the structure/activity relationship of a catalyst in a reaction. The combination of operando X-ray techniques with other analytical techniques helps to understand the kinetics of a reaction. It provides a better understanding of the electrocatalytic reaction mechanism. In the coming years, further insight will be gained in electrocatalysis by combining multiple analyses in a single experimental setup.

## Author Contributions

GN prepared the outline of the review article and guided VV in preparing the first draft to final version. VV prepared the first draft of the review article and made the corrections in the final version as suggested by GN.

### Conflict of Interest

The authors declare that the research was conducted in the absence of any commercial or financial relationships that could be construed as a potential conflict of interest.
